# A Medical Degree for Thirty-five Dollars

**Published:** 1896-12

**Authors:** 


### A MEDICAL DEGREE FOR THIRTY-FIVE DOLLARS.

The following lithographed letter is being sent broadcast to physicians who do not appear as graduates in medicine in Polk's Medical Directory:

FRED. RUTLAND, M.D., Pres't. A. NEVE RUTLAND, M.D., Sec'y.
CHAS. PODMORE, M.D., Treas.
Incorporated under the laws of the State of Wisconsin.
WISCONSIN ECLECTIC MEDICAL COLLEGE,
OF MILWAUKEE, WIS.
CORRESPONDENCE DEPARTMENT,
1001 W. Congress St.

CHICAGO, ILL., November, 1896.

DEAR DOCTOR:—We notice your name in a Medical and Surgical Directory, but with a * appended. This usually means (although not necessarily so) that the person so designated is not a graduate of a Medical School, and has no diploma. If, however, it should be that you are a graduate, and have a regular diploma, then we can but tender our most sincere apologies for troubling you on the matter. But, on the other hand, if you are not a graduate, and have no regular diploma, then the perusal of the enclosed prospectus cannot fail to be of the most primary importance and interest to you. We would also desire to draw attention to the fact that to Practising Physicians our fees are much reduced from the regular rates. To this class our fees are $35.00, all inclusive.

As proof of our legal standing and right to confer the degree of M.D., we can supply *Certified* copies of our charter at 25 cents each, simply covering the cost of certifying-officer's fee.

Trusting soon to hear from you, and standing ready to answer any or all questions you may wish to submit, we are yours very sincerely,

WISCONSIN ECLECTIC MEDICAL COLLEGE,
FRED. RUTLAND, PH.D., M.D.

A similar letter is sent to druggists who "have aspirations to a profession which runs side by side with that of pharmacy, if they desire to become physicians and have the legal right to append M.D. to their names." "Why," these letters ask, "should not the pharmacist avail himself of the opportunity (while he has it) to take another step on the ladder of life and become an M.D?" Persons are further assured that the "diplomas of the Wisconsin Eclectic Medical College are perfectly good in law, binding and valid."

We publish this business, of course, only to condemn it, and to assist in putting medical boards on notice for these "hand-me-down" diplomas. The existence of such an institution is a disgrace to the State of Wisconsin, which should take prompt action in having this charter annulled. It may be mentioned that the president and secretary of this Correspondence "College" are man and wife, respectively. Look out for the diplomas of Dr. and Mrs. Rutland.

